# Inflammasomes and Colorectal Cancer

**DOI:** 10.3390/cells10092172

**Published:** 2021-08-24

**Authors:** Sanaz Keshavarz Shahbaz, Khadijeh Koushki, Seyed Hassan Ayati, Abigail R. Bland, Evgeny E. Bezsonov, Amirhossein Sahebkar

**Affiliations:** 1 Cellular and Molecular Research Center, Research Institute for Prevention of Non-Communicable Disease, Qazvin University of Medical Science, Qazvin 3419759811, Iran; Sanaz.ks_023@yahoo.com; 2 Department of Immunology, School of Medicine, Mashhad University of Medical Sciences, Mashhad 9177948564, Iran; Koushki989@yahoo.com; 3 Immunobiochemistry Lab, Immunology Research Center, School of Medicine, Mashhad University of Medical Sciences, Mashhad 9177948564, Iran; ayatish@yandex.com; 4 Department of Pharmacology and Toxicology, School of Biomedical Sciences, University of Otago, Dunedin 9016, New Zealand; abigail.bland@postgrad.otago.ac.nz; 5 Laboratory of Angiopathology, Institute of General Pathology and Pathophysiology, 8 Baltiiskaya Street, 125315 Moscow, Russia; evgeny.bezsonov@gmail.com; 6 Biotechnology Research Center, Pharmaceutical Technology Institute, Mashhad University of Medical Sciences, Mashhad 9177948564, Iran; 7 Applied Biomedical Research Center, Mashhad University of Medical Sciences, Mashhad 1313199137, Iran; 8 School of Medicine, The University of Western Australia, Perth 6009, Australia; 9 School of Pharmacy, Mashhad University of Medical Sciences, Mashhad 9177948564, Iran

**Keywords:** cancer, inflammation, inflammasome, pyroptosis, colorectal cancer

## Abstract

Inflammasomes are important intracellular multiprotein signaling complexes that modulate the activation of caspase-1 and induce levels of the proinflammatory cytokines interleukin-1β (IL-1β) and IL-18 in response to pathogenic microorganisms and molecules that originated from host proteins. Inflammasomes play contradictory roles in the development of inflammation-induced cancers. Based on several findings, inflammasomes can initiate and promote carcinogenesis. On the contrary, inflammasomes also exhibit anticancer effects by triggering pyroptosis and immunoregulatory functions. Herein, we review extant studies delving into different functions of inflammasomes in colorectal cancer development.

## 1. Introduction

Inflammasomes are critical regulators of inflammation by stimulating the secretion of proinflammatory cytokines after sensing harmful pathogens and endogenous danger signals by innate immune system receptors (pattern-recognition receptor (PRR)) [1,2]. The tight regulation of inflammasomes is essential, as their deficient activation can lead to exacerbating and persistent pathogenic infections while upregulation can cause autoinflammatory disorders [3,4]. Therefore, understanding the structure of the inflammasome complex and activation/regulation mechanisms could help to modulate and manage different disorders.

Inflammasomes are multiprotein complexes that activate inflammatory caspases in response to pathogens or danger signals. The activation of caspases lead to the production of inflammatory cytokines (specifically, interleukin 1β (IL-1β) and interleukin 18 (IL-18)) and the induction of pyroptotic cell death [5]. IL-1β and IL-18 have a central role in inflammation and immunity, and it has been established that IL-1β/IL-18 activation by inflammasomes are considered the main factors in various inflammatory disorders [6,7]. Assembling this cytoplasmic complex is triggered by the activation of the PRR by pathogen-associated molecular patterns (PAMPs) and danger-associated molecular patterns (DAMPs). Receptors that are able to assemble inflammasomes include leucine-rich repeat containing proteins (NLR) family members (like NLRC4, NLRP1 and NLRP3); proteins absent in melanoma 2 (AIM2) and pyrin (MEFV) [5]. NLRs have a nucleotide-binding and oligomerization domain (NACHT), which is located in the center and takes part in the oligomerization and dNTPase activity. NLRs also have either a caspase recruitment domain (CARD) or pyrin domain (PYD) and, in a few cases, a baculovirus IAP repeat (BIR) or a leucine-rich repeat (LRR). An apoptosis-associated speck-like protein containing a CARD (ASC) is an adaptor protein that acts as a bridge between upwards inflammasome components and caspase-1. The PYD and CARD domains are the main components of ASC. The interaction between the PYD domain of ASC and the PYD of the NLR leads to the aggregation of ASC molecules and formation of ASC filaments. On the other hand, the CARD domain of ASC interacts with the CARD of the Zymogen form of caspase-1. The Zymogen form of caspase-1 matures into caspase 1 through the proteolytic reaction. The interaction of PYD with ASC leads to the recruitment of the Zymogen form of caspase-1 and the activation of caspase-1 [8]. Upon activation, caspase-1, through its proteolytic cleavage properties, stimulates the maturation of the dominant proinflammatory precursor cytokines (IL-1β and IL-18) and releases active forms of these cytokines. Additionally, caspase-1 cleaves the substrate gasdermin D into an N-terminal fragment of gasdermin D that induces pyroptosis [9,10].

ASC has a dual role associated with cancer. It has been demonstrated that ASC expression is silenced via methylation, which inhibits tumor cell apoptosis. On the other hand, as mentioned earlier, ASC is also recognized as an inflammasome complex adaptor molecule, which mediates inflammatory cytokines production (such as IL-1β and IL-18), mediating tumor-promoting functions. Therefore, ASC may perform opposing functions, promoting tumor progression by increasing inflammatory cytokines production or tumor-suppressing by provoking tumor cell apoptosis [11].

In addition to the activation of caspases and proinflammatory cytokines, the activation of inflammasomes leads to the programmed cell death pyroptosis as a gasdermin-dependent form of cell death [12]. This is the term used to describe the release of cytoplasmic components in the extracellular space by the creation of membrane pores [13]. Pyroptosis, as an inflammatory type of programmed cell death, can protect against intracellular pathogens via removing intracellular replication niches and concurrently triggering an inflammatory response [14]. The phagocytes (dendritic cells, macrophages and neutrophils); CD4+ T cells; epithelial cells; endothelial cells; keratinocytes and neurons also undergo pyroptosis [15]. The PRRs that these cells express can recognize a broad spectrum of PAMPs and DAMPs upon microbial infection. The common PRRs include Toll-like receptors (TLRs) and NOD-like receptors (NLRs) [16]. PAMPs and DAMPs as the main stimuli trigger the formation of multiprotein complex inflammasomes, which later activate the caspases to induce pyroptosis. The inflammasome-mediated pyroptosis pathway may be canonical or noncanonical, with the prior applying caspase-1-activating inflammasomes and the end utilizing other caspases [17].

In pyroptosis, unlike apoptosis, a different set of caspases, such as caspase-1/4/5 in humans and caspase-11 in mice, are activated by the inflammasome [18]. These caspases will lead to the activation of several proinflammatory cytokines and the pore-forming protein gasdermins. The pores that are formed during pyroptosis will result in cell membrane rupture and cytokine release, as well as the release of various DAMPs such as DNA, HMGB-1 and ATP outside of the cell. DAMPs recruit immune cells and sustain the inflammatory cascade in the tissue [19,20].

The canonical inflammasome pathway is a two-step process, involving the priming and activation steps (signals 1 and 2). At first, signal 1 is provided by TNF-α and IL-1 or via the sensing of PAMPs and DAMPs by TLRs or NOD1/2, which activate NF-κB. NF-κB activation will lead to the induction of pro-IL-1β and pro-IL-18 expression. Additionally, priming prepares the inflammasomes for activation through another undiscovered mechanism. The second step is followed by PAMP and DAMP sensing by NLRs (NLRP3, NLRC4, etc.) or AIM2 and pyrin via mechanisms that are not fully understood [21]. The activation of these receptors increases the assembly of the NLRP3 inflammasome, caspase-1-mediated IL-1β and IL-18 release and, subsequently, pyroptosis. A sensor protein (PRRs), an adaptor (ASC) and an effector (caspase-1) are the main components of the canonical pathway [17].

Some NLRs, like NLRP3 and NLRC4, using ASC, will interact with the Zymogen form of caspase-1, while others like NLRP1 can directly interact with caspase-1. The Zymogen form of caspase-1 activation, and its cleavage, can catalyze the proteolytic cleavage and activation of IL-1β and IL-18. Furthermore, caspase-1 activation is likely to have a direct effect on the induction of pyroptotic cell death [21].

In addition, non-inflammasome-forming PRRs like TLRs and NOD1/NOD2 are also important in pyroptosis. These receptors, via the activation of NF-κB and MAPK-signaling pathways, will upregulate inflammatory cytokine expressions (IFN α/β,TNF, IL-12 and IL-6) [22,23]. These active inflammatory cytokines will be released from the host cells. Caspase-1 also cleaves the cytosolic gasdermin D (GSDMD) to generate an N-terminal domain (GSDMD-N) and a C-terminal domain (GSDMD-C) [24]. In normal conditions, the GSDMD-C auto-represses GSDMD-N cleavage to suppress cell lysis [25]. GSDMD-N via oligomerization forms pores in the plasma membrane with an inner diameter of 10-14 nm, which facilitates potassium efflux and the release of IL-1β, IL-18 and other cytosolic components into the extracellular area. This interrupts the cellular ionic gradient, leading to an increase in osmotic pressure and, subsequently, pyroptosis [24,25]. GSDMD-N restricts the damage to normal neighboring cells by inserting itself into the inner membrane [26].

The activation of caspase-4/5 in humans and caspase-11 in mice responds to the noncanonical inflammasome pathway, which is initiated by the binding of a lipopolysaccharide (LPS) of Gram-negative bacteria directly onto these caspases. The oligomerization and activation of these caspases cleaves GSDMD to release GSDMD-N, triggering pyroptosis. This effect is seen to increase with LPS binding to the caspases [27]. Furthermore, the influx of potassium ions upon membrane permeabilization also stimulates NLRP3 activation, resulting in NLRP3 inflammasome formation and the activation of caspase-1. These processes promote GSDMD cleaving and improve the maturation and release of the proinflammatory cytokines [17].

Previous studies have revealed that the high production of IL-1β and IL-18, through the dysregulation of inflammasome activity during the microbial invasion or intestinal inflammation, plays a critical role in the pathogenesis of inflammatory bowel diseases (IBD) and colorectal cancer. However, the exact role of the inflammasome in the progression of these disorders requires more investigation [28].

IBD (ulcerative colitis and Crohn’s disease) is at an enhanced risk of colorectal cancer (CRC) development. The disease duration, stage of disease, degree of mucosal inflammation and portion of the bowel, family history, the related primary sclerosing cholangitis and age at diagnosis are the main CRC risk factors [29,30]. The development of CRC is two to six times greater in IBD patients compared to the general population. The major factor of CRC development in IBD is dysplasia, which is a neoplastic intraepithelial transformation. The inflammation induces intestinal epithelial cell (IEC) apoptosis through tumor suppressor p53 pathways; impaired signaling by p53 may be an initial step of the dysplasia progression to cancer [30].

In the following sections, we explain the various roles of inflammasomes in the progression of IBD, the regulation of intestinal inflammation, maintenance of intestinal epithelial barrier integrity and microbiota balance. Ultimately, after discussing the main function of inflammasomes, we discuss the association between inflammasome components and intestinal carcinogenesis.

## 2. Inflammasomes and Inflammatory Bowel Disease

IBD is a chronic gastrointestinal inflammatory condition that is categorized into ulcerative colitis (UC) and Crohn’s disease (CD). IBD is the result of genetic susceptibility to the dysregulation of the immune response to bacterial antigens in the gut lumen under certain environmental factors [31,32]. The intestinal innate and adaptive immune systems, the integrity of the epithelial barrier, the balance of commensal microbiota (dysbiosis) and diet are the main factors of IBD pathogenesis [33]. The symptoms of UC and CD can be similar, such as abdominal cramps, fever, bowel diarrhea with hemorrhage and/or containing mucus; however, the location and pattern of inflammation will differ. The location of inflammation in CD may appear anywhere along the digestive tract from the mouth to the anus and affects all layers of the bowel walls. In contrast, the inner lining of the colon is the only section that is affected in UC and begins in the rectum [34,35]. One of the main characteristics of UC is crypt abscesses formed by neutrophil migration through the intestinal epithelium. Contrary to this, the formation of granulomas, fissures and fistulas is the main inflammatory characteristic of CD [34,35]. Studies examining the cytokines in IBD have demonstrated that inflammatory cytokines such as IL-1β and IL-18 are correlated with active IBD, and IL-18 gene polymorphisms are associated with CD [36,37,38]. More specifically, Th2-type cytokines are involved in the pathogenesis of UC, whereas CD is correlated to Th1 and Th17 cytokines [33,34,35].

CD4 helper T (Th) cells, upon activation, differentiate into two main effector subsets (Th1 or Th2). Th1 cells mediate cellular immunity, the defense against intracellular pathogens and the development of various types of immunopathological reactions by producing proinflammatory cytokines such as interferon-g (IFN-γ) and lymphotoxin-a (LT-α). In contrast, Th2 cells mediate humoral immune responses; the defense against intestinal nematodes and the development of atopic disorders by producing cytokines (IL-4, IL-5, IL-13, IL-9 and IL-10) that modulate the proliferation and antibody class-switching of B cells [39,40].

Recent evidence in candidate-gene approach studies suggest an association between CD and the NLRP3 gene. Genome-wide association studies (GWAS) have discovered that the genetic susceptible element of IBD includes more than 70 susceptibility loci for CD and 40 susceptibility loci for UC [41,42]. It is known that the innate immune system—specifically, inflammasomes—contributes to chronic inflammatory disorder pathologies, like IBD [43].

It has been shown that the various polymorphisms of the NLRP3 gene might result in a decrease in the expression of the NLRP3 inflammasome and, subsequently, influences IBD genetic susceptibility. Briefly, single nucleotide polymorphisms (SNPs) in the NLRP3 genes (rs10733113); the C10X allele in CARD8; the Q705K allele in NALP3; CARD8; IL-18 (rs1946518 A > C, rs360718 A > C and rs187238 G > C) and CARD15/NOD2 are CD-susceptible polymorphisms of the NLRP3 inflammasome. SNPs in the NLRP3 inflammasome (rs10925019 and rs10754558) are UC-susceptible polymorphisms of the NLRP3 inflammasome [44].

Notably, Villan et al. [45] discovered that the SNP rs10733113 in the NLRP3 gene loci is potently associated with CD disease susceptibility. However, another study [46] could not replicate this association in a UK Panel. It was revealed that the lack of function of the CARD8 mutation in the NLRP3 inflammasome contributes to CD development [47,48]. Furthermore, the C10X allele in CARD8 accompanied by an Q705K allele in NALP3, together with NOD2 wild-type alleles, results in CD susceptibility in Swedish men [49]. Moreover, some of the polymorphisms of the NLRP3 effector IL-18 (rs1946518 A > C, rs187238 G > C and rs360718 A > C) were reported to be correlated with enhanced CD susceptibility [50]. In Han Chinese, the NLRP3 SNPs rs10754558 and rs10925019 could participate UC susceptibility but not to CD [51]. Furthermore, rs10754558 polymorphisms with “GG” and “CG” genotypes have been significantly associated with UC in Iranian patients [52]. The association between some of the SNPs that affect the receptors downstream of NLRP3 include IL18R1, IL1RL1, IL1R1, IL1R2 and IL1RL2, and a susceptibility to IBD was shown in a recent GWAS meta-analysis [53]. Collectively, various studies have demonstrated that the NLRP3 inflammasome represents a significant role in colitis pathogenesis, although the results are still questionable.

The inflammasome components (caspase-1, ASC and NLPR3) participate in a cell-specific regulation of the inflammasome-induced response. Data from several sources have identified that different intestinal cell types (epithelial and hematopoietic cells) express components of inflammasomes to respond against commensal microbiota and various pathogens for preventing mucosal damage and/or cause systemic disease [43].

Inflammasome expression by intestinal epithelial cells (IEC) is anticipated to be critical for intestinal immune homeostasis, mucosal immune defense, inflammation and tumorigenesis [54]. Data extracted from purified IEC, in-situ detection and cell-specific ablation have exhibited an array of inflammasome component expressions within IEC, including NLRC4, NAIP, NLRP1, NLRP6, caspase-1, caspase-4/5 (human), caspase-11 (mouse), ASC, AIM2 and IL-18 [55,56]. It has been demonstrated that the deletion of inflammasome components is associated with an enhanced susceptibility to damage and infection. Nevertheless, inflammasomes are regulated differently in IEC compared to the classic hematopoietic cells due to the individual intestinal environment. Based on the studies, the IEC produces relatively less IL-1β [57,58] and constitutively expresses IL-18 [59]. The IEC has a diverse inflammasome composition and can also secrete other possible factors besides IL-18 upon inflammasome activation, such as prostaglandin production, which has been correlated with NLRC4 activation [60].

## 3. Inflammasomes Regulate Intestinal Inflammation

Proinflammatory cytokines play a central role in carcinogenesis following chronic inflammation. In this case, IL-1 activates innate and adaptive immune cells and triggers these cells to produce additional inflammatory cytokines, chemokines and chemical mediators for infection and the injury response. Cell proliferation, repair and the healing process are the other roles of IL-1 [6]. Activated IL-1β and IL-18 induce the inflammatory pathway in myeloid and non-myeloid cell types [61,62]. Therefore, the use of chemical inhibitors of caspase-1, IL-1β and IL-18 can help to understand the exact role of these molecules in colitis pathogenesis [63,64,65,66].

On the other hand, a non-pyroptotic function of GSDMD in controlling the release of small extracellular vesicles (sEVs) contained from intestinal epithelial cells (IECs) in response to the activation of the caspase-8 inflammasome was described by Bulek et al. (2020). This study reported that GSDMD, which is activated by the caspase-8 inflammasome accompanied by Cdc37/Hsp90, recruits NEDD4 as an E3 ligase to catalyze pro–IL-1β polyubiquitination, assisting as a signal for cargo loading into secretory vesicles. It has been reported that IBD patients and those with experimental colitis showed elevated epithelial-derived GSDMD expression. Bulek et al. (2020) also showed that GSDMD deficiency considerably decreases the severity of the disease, relating to the GSDMD-mediated release of IL-1β sEVs in the intestinal inflammation pathogenesis, as observed in IBD [67].

Miguchi et al. (2016) reported that GSDMC served as an oncogene, increasing cell proliferation in colorectal carcinogenesis. Furthermore, TGFBR2 (transforming growth factor beta receptor 2) mutations, which happen in high-frequency microsatellite instability (MSI-H) colorectal cancer (CRC), upregulate GSDMC. These elevated levels of GSDMC increase the tumor cell proliferation, recommending that GSDMC may be a hopeful therapeutic target [68].

Schwarzer R. et al. (2020) demonstrated that caspase-8 and GSDMD were both needed for the development of mixed-lineage kinase-like (MLKL)-independent ileitis in mice with epithelial fas-associated with death domain (FADD) deficiency [69].

NLRP3 is a main component of the NLRP3 inflammasome, which is activated by microbial pathogen-derived components, external ATP, crystals of uric acid and calcium pyrophosphate dihydrate and synthetic purine-like compounds [70,71,72]. Of course, it is worth mentioning that the listed stimuli for NLRP3 are not exhaustive. Different mechanisms play key roles in activating the NLRP3 inflammasome. The large variety of microbial products, infections-correlated stimuli, comprising an elevation in extracellular osmolarity or pH alterations, extracellular ATP, pore-forming toxins, β-amyloid fibers, K^+^ efflux, reactive oxygen species (ROS), activation and deubiquitination of cathepsin and extracellular matrix component degradation, molecules that are derived from the environment and host-like monosodium urate crystals and asbestos particles can increase NLRP3 inflammasome oligomerization and the activation of downstream pannexin-1 and the P2X7 receptor [73,74,75,76,77]. The major mechanisms involving NLRP3 activation are the K^+^ and Na+ cell membrane permeations. Prominently, the cytosolic K^+^ concentration drop has been shown to be adequate to activate NLRP3, whilst an elevation in cytosolic Na+ was modulated but not necessarily needed for inflammasome activation [78].

**Figure 1:**
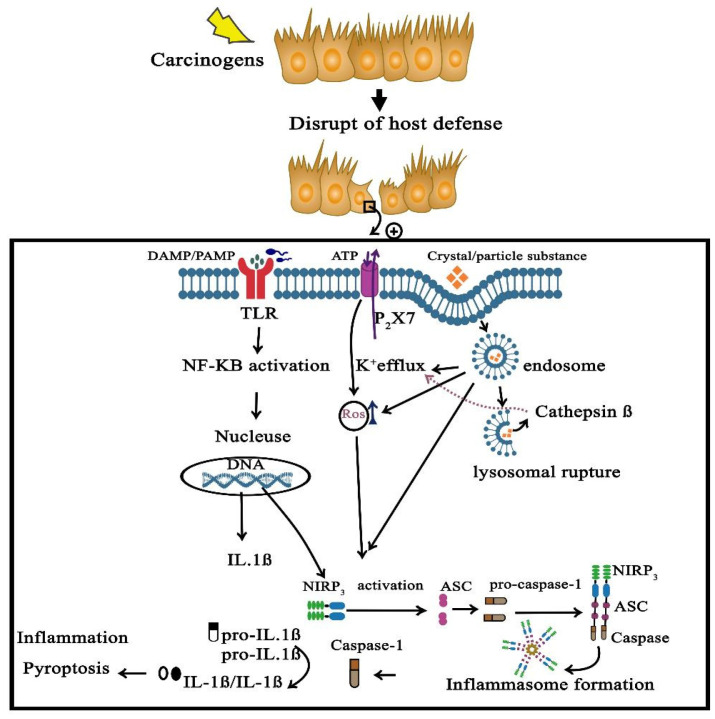
Inflammation-induced intestinal carcinogenesis through the activation of the NLRP3 inflammasome. Carcinogens can disrupt the host defense through an increase in ROS/RNS production that results in the disruption of immune barriers. NLRP-3 activation is the main mechanism of inflammation-induced intestinal carcinogenesis. NLRP-3 activation is mediated through several approaches. DAMPs/PAMPs (pathogens) are recognized by TLRs that activate the NF-κB pathway to enhance the translation of proinflammatory cytokines. ATP (as another stimulus) will open the P2X7 (ATP-ion channel), which leads to K^+^ efflux, also activating the NLRP3 inflammasome directly. Crystals and particle substances will be transported through endosomes and, after the merge with lysosomes, will result in lysosomal rupture and directly induce a K^+^ efflux and NLRP3 inflammasome formation. NLRP3 inflammasomes comprised of NLRP3, ASC and caspase-1 will induce the cleavage of pro-IL-18 and pro-IL-1β into their mature active forms through activated caspase-1, which produces inflammation and pyroptosis. DAMPs, damage-associated molecular patterns; PAMPs, pathogen-associated molecular patterns; ROS, reactive oxygen species; RNS, reactive nitrogen species.

The interaction of pannexin-1 and the P2X7 receptor induce the opening of a large pore permeable to hydrophilic macromolecules in the cell membrane [79]. P2X7 receptors are danger signal receptors in immune cells involved in various functions, such as the production and release of proinflammatory cytokines and apoptosis [80]. Moreover, intestinal epithelial and immune cells express P2X7 receptors, suggesting that purinergic signaling acts as an innate immune system component involved in inflammation control and cell survival in the gut and gut-associated lymphoid tissues [81]. Furthermore, P2X7 receptor expression on intestinal epithelial cells is upregulated by interferon, which indicates a Th-1 immune response [82]. ATP, through the production of ROS following P2X7 receptor activation, induces apoptosis and autophagy in human epithelial cells [83]. Collectively, these findings confirm that the activation of the P2X7 receptors/pannexin-1 and the sequential NLRP3 inflammasome plays a central role in the dysregulation of the immune response during pathogenesis of diseases such as IBD (Figure 1).

NLRP3 inflammasome deficiency, which comprises a deficiency of ASC, caspase-1 and NLRP3, results in a susceptibility to dextran sodium sulfate (DSS)-induced colitis, characterized by symptoms of colonic injury and enhanced inflammation [84,85,86,87]. Furthermore, NLRP3 mutations have been shown to correlate with CD susceptibility [45]. This suggests that NLRP3 could be protective against IBD. IL-1β and IL-18 are other inflammasome substrates that have been proposed to have a protective role in IBD [36,37,88]. Intestinal chronic inflammation, such as IBD, has been known to develop into colorectal cancers. Several studies have reported that NLRP3 inflammasome-deficient models correlate with an increase in the risk of colorectal cancer [84,85,89]. For example, Allen et al. (2010) examined NLRP3 or caspase-1-deficient (*Nlrp3*^−/−^ or *Casp1*^−/−^) mice, using an azoxymethane plus DSS (AOM + DSS) model. The authors reported that the deficient mice had a greater predisposition toward colorectal tumorigenesis and a lower survival rate compared to wild-type mice [84,85,89].

However, other studies do not support this research, whereby two studies failed to confirm the protective role of NLRP3 against DSS-induced colitis [90,91]. Hu et al. (2010) reported that only caspase-1-deficient mice were susceptible to colitis-associated adenomatous polyp formation in the gut. However, NLRP3 knockout mice had no change in tumor formation or tumor frequency compared to wild-type mice [91]. This inconsistency may be due to the presence of different gut microflora contents in animals or differences in experimental protocols. Other members of NLR could also regulate inflammasome activation during colitis. In this regard, studies have reported that NLRC4 and NLRP6 play the main role in protection against colorectal tumorigenesis [91,92]. Although Hu et al. (2010) found no change between the tumors of wild-type and *Nlrp3*^−/−^ mice, the authors did report an increase in tumor load and tumor frequency of *Nrc4*^−/−^ mice [45]. Therefore, inflammasome components and related cytokines have been shown to take part in the modulation of intestinal homeostasis.

## 4. Inflammasomes Protect the Integrity of the Intestinal Epithelial Barrier

In this section, we focus on the function of inflammasomes in the protection against colitis and colorectal cancer. Based on recent evidence, NLRP3, NLRP6 and caspase-1-deficient mice were significantly more sensitive to dextran-FITC, a polysaccharide that is toxic to the epithelium of the colon, than wild-type mice [85,87,92]. In fact, provoking a cytotoxic attack (using a compound like dextran-FITC, as seen in the last studies) to the intestinal epithelium has been seen to induce repair responses identified by enhanced stem cell division at the base of the crypt to renew the injured enterocytes [93]. In this regard, IL-1β and IL-18 also participate in repairing the damaged intestinal epithelium [94]. In order to confirm these findings, Zaki et al. (2010) used NLRP3 and caspase-1-deficient mice treated with dextran-FITC. The authors reported that the wild-type and deficient mice all increased the epithelial cell proliferation during acute colitis; however, when comparing the groups, the deficient mice presented with a decrease in proliferation when compared to the wild-type. This indicates that inflammasomes are required for repairing damaged tissue [87,92].

Additionally, inflammasome activation in myeloid cells has several outcomes. NLRP3 is originally expressed in macrophages, monocytes, dendritic cells (DC), granulocytes, osteoblasts and epithelial cells. The expression of NLRP3 in myeloid cells is profoundly inducible. Myeloid cells like macrophages, DC and granulocytes, in addition to lymphoid cells (T cells), express NLRP6. Pending development, NLRP6 is activated by PPAR-γ in the intestinal epithelium. Studies have discovered that NLRP6 plays a role in protecting against experimental colitis and colitis-associated cancer and monocytes [95]. The evaluation of antitumor immunity via DC vaccination demonstrated that the expression of NLRP3 in the tumor microenvironment promoted the migration of tumor-associated myeloid-derived suppressor cells (MDSCs) to the site of the tumor. Furthermore, the increased expression of NLRP-3 in MDSCs following this treatment was able to overcome the antitumor immunity [95]. A related study reported that Nlrp3-deficient mice also have fewer MDSCs accumulating at the region of the tumor and improved survival upon DC vaccination [96].

Bone marrow chimera studies have confirmed the function of inflammasomes in maintaining the integrity of the epithelial barrier, as well as protection against colitis and colorectal tumorigenesis. These studies have shown that acute colitis could be prevented by inflammasome activation in nonhematopoietic cells [84,92]. On the other hand, inflammasome activity in myeloid cells is critical for the suppression of polyp formation during chronic inflammation. This was reported by Allen et al. (2010), who used the AOM plus DSS tumorigenesis model and found that mice lacking caspase-1 and ASC had an increase in polyp formation, morbidity and disease outcome [84,92]. From these results, it can be deduced that intestinal epithelial cells activate inflammasomes during the acute phase of colon inflammation to repair the epithelial barrier and maintain the homeostasis of the gut epithelium. Myeloid cells may suppress the production of carcinogenic factors, which can provide a susceptible tumor microenvironment during the chronic phase of inflammation. Therefore, inflammasome activation in different cells and tissues could modulate inflammation and take part in tissue repair to prevent carcinogenic development in various stages of the disease [97].

In addition, other components of inflammasomes have recently been investigated, such as the NLR family of apoptosis inhibitory proteins (NAIP) (NAIP1, 2, 5 and 6 in mice and hNAIP in humans) and NLRC4. Recent studies have discovered that NAIPs and NLRC4 in intestinal epithelial cells respond to several stimuli, such as enteropathogenic bacteria invasion [98,99] and carcinogen exposure [91,100]. The main role of NAIPs is recognizing Gram-negative bacteria components such as flagella and type-three secretion systems (TTSS) and activate the NLRC4 inflammasome [101]. There is a strong correlation between NLRC4 and the function of phagocyte cells in order to eliminate the mucosal pathogen loads in the intestine lamina propria and other areas, the restriction of adverse pathology, systemic bacterial transmission and intestinal tumorigenesis [102,103,104]. Furthermore, NLRC4 has a protective role against acute mucosal infection by enteric bacteria like *Salmonella typhimurium* and *Citrobacter rodentium* [98,99]. NLRC4, through recruiting and activating the zymogen form of caspase-1, led to pro-IL-18 maturation [105]. IL-18 is a proinflammatory mediator that regulates proinflammatory responses in intestinal diseases, and its production may be a principal etiological factor for IBD patients [106,107].

Sellin et al. (2014) reported that epithelial NAIP/NLRC4 is involved in the removal of pathogen-infected intestinal epithelial cells from the mucosa during early infection. The authors also discovered that caspase-1 plays an important role in NAIP/NLRC4 defense, using a *caspase-1*^−/−^-deficient mouse model. Without caspase-1, the inflammasome could not function correctly to remove the infected intestinal epithelial cells and provide protection from infection [98].

NAIP/NLRC4 inflammasomes participate in antimicrobial responses in intestinal epithelial cells by inducing nitric oxide production, intracellular trafficking management and actin cytoskeleton reorganization [108,109,110,111,112]. Intestinal epithelial cell inflammasomes are activated through carcinogen exposure and chronic inflammation and go on to decrease adenoma development in the colonic epithelium and, further, colorectal carcinoma development. Thus, epithelial NLRC4 and NAIPs could prevent the development of colon tumorigenesis [91,100].

## 5. NLRP6 Inflammasomes Maintain the Microbiota Balance

Intestinal epithelial cells are also involved in the maintenance of gut microbiota and modulation of the nutrient requirements for commensal organisms that secrete mucus and antimicrobial peptides to limit pathogen colonization. The dysregulation of this balance leads to dysbiosis, the outgrowth of “pathobionts” and increases the predisposition of a disease [113]. It is suggested that NLRP6, as one of the inflammasome components, is involved in maintaining the healthy microbiota in intestinal epithelial cells. It is likely that NLRP6, through an interaction with ASC, forms a canonical inflammasome [114]. NLRP6 is predominantly expressed in intestinal epithelial and goblet cells throughout the small and large intestines and nonepithelial cells of the gut mucosa (myofibroblasts and myeloid cells) [92,98,115,116,117].

The previously mentioned studies reported that NLRP6 deficiency leads to exacerbated DSS-induced colitis, as well as tumor progression in response to AOM/DSS. However, these studies could not explain the exact mechanism of NLRP6 for these phenotypes [92,115,116]. Other studies in this field have described that the protective effect of NLRP6 could be via the induction of IL-18 production by intestinal epithelial cells [115], hematopoietic cells [92] or lamina propria myofibroblasts by NLRP6 having an effect on intestinal epithelial cell proliferation and self-renewal [116]. Interestingly, colitis and carcinogenesis susceptibility with NLRP6, ASC or IL-18-deficient mice could be transferred to naive wild-type mice by cross-fostering and cohousing the mice together, leading to a dysbiotic gut microbiota [115,118]. However, the mechanisms of NLRP6 signaling that manage the gut microbiota conditions and inhibit the pathological drift are poorly understood. One study revealed that *Nlrp6*^−/−^ mice have central defects in goblet cell granule release and a compromised mucus layer that correlates to autophagy defects [117]. Therefore, there is evidence that NLRP6 serves to improve the secretory activity of intestinal epithelial cells and mediates the release of IL-18 through other cell types.

## 6. Inflammasome and Intestinal Tumorigenesis

This section will review the latest studies that examine the correlation between inflammasomes and intestinal tumorigenesis and further discuss the contradictory roles of the inflammasome (Table 1).

Colorectal cancer develops when tumor cells succeed to escape from both cell-intrinsic and cell-extrinsic cancer-inhibited mechanisms [119,120]. Several cancers prevent the antitumor immune response through the induction of low levels of inflammation, and hence, chronic inflammation could induce the development of tumor cells. The innate immune system prime adaptive immunity induces an inflammatory response that could eradicate tumor cells. IBD, as an example of an inflammatory disease, could progress to colorectal cancer [121,122].

**Figure 2:**
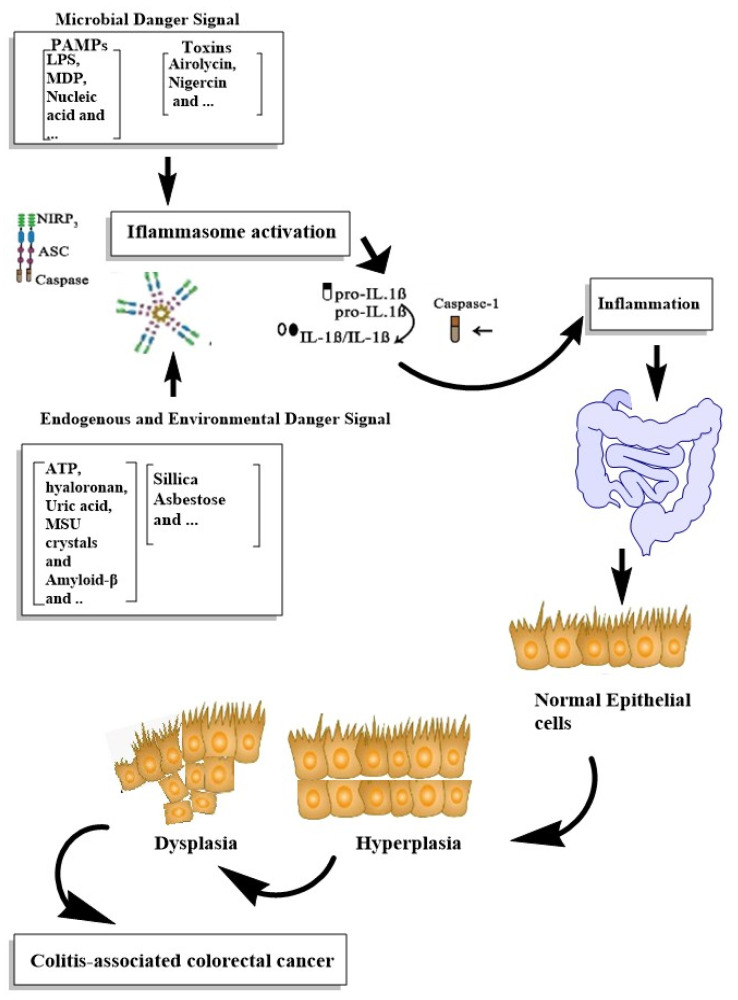
Summarizing the roles of inflammasome activation in IBD progression to CRC.

Inflammasomes, as the main participants of inflammation, were considered to be associated with the modulation and progression of tumors. The remarkable epithelial responses, such as the proliferation of intestinal epithelial cells and tissue repair; chronic immune inflammatory responses, such as increased levels of cytokines, chemokines and ROS production and alterations in the intestinal microbiota content, were associated with colorectal tumor progression [121,123]. The main stimuli of inflammasome activation and its role in the IBD progression to CRC are demonstrated in Figure 2.

The azoxymethane/dextran sulfate sodium (AOM/DSS) model is often used to assess the role of inflammasomes in the progression of intestinal tumors. Tanaka et al. (2003) found that administering DSS following a low dose of AOM produced a tumor-promoting inflammatory response in the colon of mice [124]. Using this model, a series of studies reported that inflammasome components are essential for protecting mice from colitis-associated colon cancer by also using NLRP or caspase-1-deficient mice. Zaki et al. (2010) used NLRP3, ASC and caspase-1-deficient mouse models to demonstrate that the lack of NLRP3 inflammasome components results in the development of AOM/DSS-induced inflammation and a significant progression of colorectal cancer. The authors also reported that reduced IL-18 levels, as a consequence of lacking these components, led to the impaired production and activation of IFN-γ and tumor-suppressor STAT1. Therefore, IL-18 signaling downstream of the NLRP3 inflammasome plays a central role in the protection against colorectal cancer progression [89]. NLRP6 deficiency has also been shown to dysregulate the renewal of colonic mucosa, proliferation and organization of epithelial cells through casein kinase I isoform epsilon (Csnk1ε) and SMARRC1 upregulation. This resulted in accelerated colitis-associated tumor growth, further suggesting that NLRP inflammasomes play a crucial role in the prevention of colorectal cancer [116].

NLRP6 deficiency in intestinal epithelial cells leads to a decrease of the IL-18 levels and altered fecal microbiota. Intestinal hyperplasia, inflammatory cell migration and an increase in the severity of DSS-induced colitis were some of the characteristics of NLRP6-deficient mice. The NLRP6 deficiency led to a decrease in intestinal epithelial IL-18 production, which produced enhanced colitogenic features in the microbiota. These microbiota alterations resulted in the upregulation of CCL5 production, immune cell recruitment and an increase in inflammation [115]. Using the AOM/DSS-induced colitis-associated colorectal cancer model, Hu et al. (2013) demonstrated that NLRP6 and ASC-deficient mice had a colitogenic gut microflora that induced exacerbated inflammation-induced colorectal cancer. During colonic inflammation, IL-18 induces the aberrant microflora that stimulates CCL5 production, promoting epithelial cell proliferation through local activation of the IL-6 pathway. Consequently, the susceptibility of colon tumor progression in these NRLP6 and ASC-deficient mice was enhanced [118]. Similar to Hu et al. (2013), Chen et al. (2011) demonstrated that NLRP6-deficient mice exhibited enhanced inflammation within the colon after DSS administration, and these mice were more susceptible to DSS-induced colitis such as colorectal tumorigenesis. With the lack of NLRP6, the mice had an impairment in the function to reduce inflammation and repair injured epithelium. This resulted in a continued proliferation of epithelial cells, and consequently, this process led to cancer progression in these mice during chronic inflammation [92].

Similar to the previous studies, Allen et al. (2018) confirmed that NLRP-3, Pycard and caspase-1-deficient mice had an increase in the risk of developing acute and recurring colitis and colorectal cancer. This was also accompanied by diminished levels of IL-1 and IL-18 at the tumor area. What is different with this study is that Allen et al. (2018) reported no change in disease improvement or outcome with NLRC4 deficiency [84]. Dupaul-Chicoine et al. (2015) also demonstrated that the lack of NLRP3 inflammasome components resulted in the impairment of IL-18 signaling, which led to exacerbating the metastatic growth of liver colorectal cancer. The anticancer function of the adaptive immune system (B and T cells) and gut microbiota did not influence the tumor growth. Alternatively, the inflammasome/IL-18 signaling affected hepatic natural killer cell maturation, surface expression of the death ligand FasL and the potential to eliminate FasL-sensitive tumors [125].

Other research has confirmed that caspase-1, as an inflammasome component, is associated with mucosal tissue repair (crypts and surface epithelia) through the prevention of severe stimulation of lamina propria immune cells via luminal bacteria and limiting the chemotactic factors production. Caspase-1 is also necessary for regulating the interactions between host tissues and the luminal microbiota by modulating the numbers of commensal bacteria that flow into sites of colonic damage. Furthermore, caspase-12 deficiency was correlated with colorectal tumor promotion and progression due to an elevated expression of tumor-promoting genes like Bloom Syndrome gene (Blm) [85]. Caspase-1 deficiency has been demonstrated to enhance tumor development in an AOM/DSS-induced colitis-associated colorectal cancer model through the regulation of colonic epithelial cell proliferation and apoptosis that is usually mediated by NLRC4 activity [91].

Man et al. (2015) and Wilson et al. (2015) further examined the inflammasome component, AIM2, and how a knockout of this protein effects colorectal formation. AIM2-deficient mice exhibited intestinal stem cells that were predisposed to uncontrolled proliferation, which made them prone to colon tumor development through independent inflammasome mechanisms. This defect led to aberrant Wnt signaling that resulted in the development of a population of tumor-generating stem cells. Host genetic factors and gut microbiota also had a synergistic effect on the susceptibility to colorectal cancer in AIM2-deficient mice [126]. It has also been reported that AIM2 deficiency in a AOM/DSS mouse model of colorectal cancer resulted in an increase in tumor load in an inflammasome-independent manner (Akt activation) and was mediated by a non–bone marrow source of AIM2 [127]. Another study conducted by Karki et al. (2016) discovered that AIM2-deficient mice had a greater susceptibility to colonic tumor development through aberrant Wnt signaling that developed an uncontrolled proliferation of tumor-initiating intestinal stem cells [128]. Chen et al. (2017) reported that AIM2 acts as a tumor suppressor by blocking G1-to-S-phase cell cycle transition and suppressing the phosphatidylinositol 3-kinase (PI3K)/protein kinase B (Akt) pathway [129]. Therefore, AIM2 modulation could be applied as a therapeutic approach for preventing colorectal cancer.

Liu et al. (2015) analyzed the gene expression of NLRC3, NLRC4, NLRC5, AIM2, NLRP1, NLRP3, NLRP6, NLRP12, NOD1 and NOD2 by combining a bioinformatic analysis (ten public colorectal cancer datasets from the Oncomine^®^ Platform) and experimental, verifying utilizing clinical tissue samples during a cohort study. They found that the mRNA expression of NLRC3 as an inflammation checkpoint; NLRP1, NLRP3 and NLRC4 as the components of inflammasome and AIM2 were all decreased in colorectal cancer. NOD1 and NOD2 expression were enhanced in colorectal cancer, and NLRC5, NLRP6 and NLRP12 had no significant changes compared to the controls. Moreover, ASC and caspase-1, as components of the inflammasome, and the downstream substrates of caspase-1; IL-1β and IL-18 were decreased in colorectal cancer cells. The authors also reported that the reduction in NLRC3 and AIM2 mRNA expression in colorectal cancer cells was correlated with the progression of colorectal cancer. Therefore, NLR and AIM2 genes can be used as biomarkers of colorectal cancer and cancer progression [130].

NALP1 has also been explored, whereby Chen et al. (2015) examined NALP1 expressions in human normal and malignant colon tissues using a microarray assay, Western blotting and RT-PCR and explored the NALP1 expression in different cell lines and animal models of colon cancer before and after treatment with DAC (5-aza-2-deoxycytidine), an antitumor drug. They demonstrated that human colorectal tumoral tissues expressed low levels of NALP1 compared to peritumoral tissues and were correlated with the survival and tumor metastasis of patients. They also reported that DAC was able to increase the expression of NALP1 in vitro and in vivo and increased the survival rates in mice [131].

NLRP3 has a different function in the regulation of tumorigenesis when compared to other components in the inflammasome. Studies have shown that NLPR3 helps the progression of tumorigenesis via the elevation of inflammation [132,133].

Du et al. (2016) examined mice treated with AOM and high dietary cholesterol and discovered that the treated mice had an increase in colorectal tumorigenesis. The authors determined that this was via an induction of the NLRP3 inflammasome, the formation of a NLRP3–ASC–caspase-1 complex assembly and an increase in IL-1β production. In fact, cholesterol produces higher levels of mitochondrial ROS by inhibiting AMPKα in macrophages, which leads to the activation of the NLRP3 inflammasome, resulting in the activation of β-catenin signaling. Subsequently, a deletion of NLRP3 in AOM-treated mice resulted in a decrease in the secretion of IL-1β [132].

Interestingly, inflammasome-independent NLRP3 has also been demonstrated to develop TGF-β-induced epithelial–mesenchymal transition (EMT) in colorectal cancer. Wang et al. (2016) reported that NLRP3 was significantly overexpressed in mesenchymal-like colon cancer cells and, further, that TNF-α and TGF-β induced NLRP3 upregulation during EMT in colon cancer epithelial cells. NLRP3 knockdown in colorectal carcinoma cells maintained the epithelial spindle-like morphology and decreased cell migration and invasion. This was determined to be the result of a decrease in vimentin, MMP9 and Snail1 and an increase in E-cadherin expression. Finally, Wang et al. (2016) found that TNF-α or TGF-β-induced EMT was regulated by NF-κB signaling, not through inflammasomes [134].

Deng et al. (2019) discovered that colorectal cancer tissue was surrounded by macrophages that expressed high levels of NLRP3. The NLRP3 inflammasome was also activated in macrophages through the macrophage–colorectal cancer cell crosstalk that resulted in the accelerated migration of colorectal cancer cells and improved their metastatic ability [135]. Chung et al. (2019) also reported that NLRP3-mediated colitis and inflammation-associated colon carcinogenesis could be attenuated by a pretreatment with inactivated probiotic *Enterococcus faecalis* or NLRP3 siRNA. *Enterococcus faecalis* can suppress NLRP3 inflammasome activation in macrophages following the reduction of caspase-1 activation and IL-1β maturation. Indeed, *Enterococcus faecalis* attenuated phagocytosis, which is required for the complete activation of the NLRP3 inflammasome in response to commensal microorganisms [136].

As previously mentioned, NRLP3 can promote colorectal cancer development; therefore, Zhao et al. (2018) evaluated therapeutic effects of a small-molecule AMP-activated protein kinase activator (GL-V9) in a colitis-associated colorectal cancer model. GL-V9 was found protected against colitis and tumorigenesis via triggering autophagy to degrade the NLRP3 inflammasome [133].

NAIP1-6 are other components of the inflammasome, which have also been reported to be main regulators of colorectal tumorigenesis. NAIP1-6-deficient mice, but not NLRC4-deficient mice, had enhanced STAT3 activation and p53 inhibition after carcinogen exposure in an epithelial-intrinsic manner. NAIP1-6-deficient mice also exhibited increased colon tumorigenesis in an inflammation-independent model of colorectal carcinoma. Therefore, NAIPs are also considered to have a role in the protection against colon tumorigenesis progression through an increase in the elimination of carcinogen-elicited epithelium [100].

NLRP12 is derived from both hematopoietic and nonhematopoietic origins and participates in inflammation but, over time, mostly inhibits tumorigenesis through negative regulation of the ERK and AKT-signaling pathways in tumor tissues. This aberrant activation in ERK and AKT led to the inhibition of TRAF3 degradation, activation of NIK and p100 processing to p52. Consequently, NLRP12-deficient mice were highly susceptible to colitis and colitis-associated colorectal cancer through the increase in noncanonical NF-κB activation and expression of Cxcl13 and Cxcl12 as target genes that were associated with cancer [137].

Finally, Bilonda et al. (2018) demonstrated that inflammasomes of colorectal cancer cells are maintained and participate in the Th1/Tc1 antitumor response elicited via tumor-infiltrating T lymphocytes (TILs). These TILs express various immune checkpoints, including PD1 and TIGIT, that likely lead to their exhaustion. So, the modulation of inflammasomes of tumor cells towards enhancing the Th1/Tc1 immune response in relation to immune checkpoint blockers could serve as a potential novel therapeutic target in colorectal cancer [138].

Recently, it has been demonstrated that NLRP6 and IL-18, alone or in combination, are powerful predictors of patient outcomes in colorectal disease, and examining the epithelial inflammasomes may improve clinical decisions for more reliable prognostic evaluations and recognize novel therapeutic purposes in CRC. Moreover, research has reported that re-establishing normal NLRP6 expression in tumor cells may present therapeutic advantages over preventing progress towards advanced CRC [139]. The aberrant expression of NLRP3 was reported in CRC, and high NLRP3 expression levels led to a poor prognosis for patients with CRC. Therefore, targeting NLRP3 and the NLRP3–MAPK–mTOR–S6K1 axis may be promising for the targeted therapy of CRC, particularly for patients with a resistance to mTORC1-targeted therapies [140]. Mardani et al. (2021) showed that activation of the NLRP3 inflammasome contributed to the development and progression of colorectal cancer, and this activation correlated with the progression of the epithelial–mesenchymal transition (EMT) process [141]. Furthermore, another study by Shao et al. (2020) reported that NLRP3 was remarkably upregulated in human CRC tissues, which was associated with a greater tumor size, metastasis, invasion and poor tumor staging. The authors demonstrated that NLRP3 likely regulates CRC metastasis by activating the EMT program and is a potential therapeutic target [142]. Furthermore, a study by Mutala et al. (2021) reported that tumor cells of most CRCs display a functional and activated caspase-1/IL-18 axis that can modulate the IFN-γ response elicited by the Th1/Tc1 response. Moreover, the authors strongly suggested that targeting the inflammasome pathway could improve the antitumor immune response in subgroups of CRC [143]. Another study reported that the inhibition of NLRP3 inflammasome assembly in macrophages was responsible for the preventive effect of arctigenin against CAC in patients with colitis [144].

Collectively, one of the main predisposing factors of CRC is inflammation [145]. Evidence suggests that inflammasomes take part as a double-edged sword in developing or alleviating intestinal tumorigenesis. The understanding of the inflammasome components, biology and function in different cells/tissues and discovering the various interactions of inflammasomes with different inflammatory disorders could help to modulate/treat them.

## 7. Conclusions

Inflammasomes play several roles in the defense against pathogens, mediating colorectal tumor induction and progression by creating a proinflammatory microenvironment (composed of cytokines, chemokines and ROS). This microenvironment induces malignant transformation characterized by intestinal proliferation of the epithelial cells and tissue repair and alterations in the intestinal microbiota that overcome the local immunity generated by natural killer or T cells. On the other hand, inflammasomes may also have an anticancer role. Indeed, multiple factors such as tumor microenvironments and gut microbiota could affect the exact role of an inflammasome. Determining the exact activation mechanisms of NLRP1, NLRP3 and NLRC4 (as the main components of the inflammasome) and the regulator molecules of the inflammasome pathway, the effect of the inflammasomes on different cell types and host antitumor immunity and immunotherapy during tumor growth and progression could be accomplished by evaluating the dysregulation or mutation of inflammasome components, using animal models or cancer cell lines. Several studies have shown that NLRP3/NLRC4-deficient mice were more susceptible to colorectal cancer progression due to the suppression of tumor suppressor genes such as p53 and the overexpression of oncogenes such as Wnt. Therefore, inflammasomes might serve as potential biomarkers of the tumor microenvironment status and be employed for cancer diagnosis or prognosis. Collectively, the development of our knowledge about the main role of inflammasomes in cancer progression and the downstream signaling pathways may provide novel insights for designing new preventive and therapeutic approaches against cancer.

## Figures and Tables

**Table 1:** The role of inflammasomes and their respective mechanisms in colorectal cancer.

Inflammasome Components Mice Model/Human	Suggested Mechanisms	Clinical Outcomes	References
NLRP3, ASC, Casp-1 and IL-18-deficient mice	-IL-18 levels ↓ -The production and activation of IFN-γ and tumor suppressor STAT1 ↓	-Elevated susceptibility of DSS-induced colitis and colorectal cancer progression	[89]
NLRP6-deficient mice	-Csnk1ε that stabilizes β-catenin↑ -SMARRC1↑	-Enhanced susceptibility to experimental colitis and colorectal tumorigenesis -Develop relapsing colitis -Decreased regeneration of the colonic mucosa (Wound Healing) -Enhanced epithelial cell organization and proliferation upon Injury -Enhanced tissue injury with bleeding and ulcerations -Decrease of diseased colon length -Decreased body weight of mice	[116]
NLRP6-deficient mice	IL-18↓, CCL5↑	The intestinal hyperplasia, inflammatory cells migration, and increasing of the severity of DSS-induced colitis	[115]
NLRP6 and ASC-deficient mice	IL-18, ↓ CCL5, IL-6↑	Enhanced susceptibility to colorectal tumorigenesis	[118]
NLRP3, NLRC4, Pycard and casp-1-deficient mice	Pycard and casp-1: IL-1β and IL-18 levels↓	-Nlrp3, Pycard, and casp-1 deficiency: Enhanced acute and recurring colitis and colorectal cancer progression -NLRC4-deficient mice didn’t show significant differences in disease progression or outcome	[84]
NLRP3, NLRC4 and Aim2-deficient mice	IL-18 ↓, FasL↓	Colorectal cancer metastatic growth in the Liver	[125]
Nlrp6-deficient mice	IL-18 ↓ proinflammatory cytokines (TNF, IL-6 and IL-1β) ↑	-Elevated susceptibility to colitis and colitis-associated colon tumorigenesis -significant inflammation and injury within the colon -Prolonged epithelial cells proliferation	[92]
Casp-1 and Casp-12-deficient mice	IL-18 ↓ Nf-kB ↑	-Decrease of tissue repair -Increase of colitis, and colitis-associated tumorigenesis	[85]
Casp-1 and NLRC4-deficient mice	Colonic epithelial cell proliferation and apoptosis ↑	-Increase the colon tumorigenesis	[91]
Aim-2-deficient mice	Aberrant Wnt signaling	-Elevated susceptibility to colon tumorigenesis	[126]
Aim-2-deficient mice	DNA-PK and Akt activation ↑	-Elevated susceptibility to colon tumorigenesis	[127]
Aim-2-deficient mice	Aberrant Wnt signaling	-Elevated susceptibility to colon tumorigenesis	[128]
HCT116 CRC cells	Blocking of cell cycle transition from G1 to S phase and suppressing the (PI3K)/protein kinase B (Akt) pathway	- As a tumor suppressor could apply as a potential therapeutic target for future improvement of AIM2-based gene therapy for human CRC	[129]
Public CRC datasets/CRC patients	NLRC3, NLRC4, NLRP1, NLRP3 and AIM2 ↓NOD1/NOD2 ↑	-NLRC3 and AIM-2 correlated to colorectal cancer progression	[130]
Nude mice/ Colonic cancer patients, Cell culture of human colon cancer cell lines	-colonic cancer tissue: NALP-1 ↓	Elevated susceptibility to colon tumorigenesis	[131]
NLRP3-deficient C57BL/6 mice	IL-1 ↓	-NLRP3 deficiency decreased cholesterol effects in progressing of colorectal tumor	[132]
Cell culture of human colon cancer cell lines	vimentin, MMP9 and Snail1 ↓ E-cadherin ↑	-Inflammasome-independent NLRP3 develop the TGF-β-induced EMT (epithelial–mesenchymal transition) in colorectal cancer	[134]
NLRP-3 suppression		-Suppression of migration of CRC cells and metastatic ability	[135]
NLRP-3 suppression	-Caspase-1 activation and IL-1β maturation ↓ -Phagocytosis ↓	-The attenuation of NLRP3-mediated colitis and inflammation-associated colon carcinogenesis	[136]
NLRP-3 degredation	triggering autophagy after a small-molecule AMP-activated protein kinase activator (GL-V9) administration ↓	-protection against colitis and tumorigenesis in colitis-associated colorectal cancer	[133]
NAIP1-6-deficient mice	STAT-3 activation ↑ P53 activation ↓	Elevated susceptibility to colon tumorigenesis	[100]
NLRP12	TRAF3 degradation, activation of NIK and p100 processing to p52 ↓ NF-KB pathway ↑ Cxcl13 and Cxcl12 ↑	Enhanced susceptibility to experimental colitis and colorectal tumorigenesis	[137]
CRC patients	-Epithelial protein expression of inflammasome components (NLRP1,3,6, ASC, AIM-2, caspase-1) and IL-1β and IL-18 -Epi IL-18 is correlated with higher lymphocyte infiltration within colorectal tumors	-NLRP6 and IL-18 expressions in epithelial tumor cells are strong predictors of patients’ outcomes -Epithelial expression of NLRP6 and IL-18 are correlated with tumor evolution -Re-establishing normal NLRP6 expression in tumor cells may present therapeutic advantages over preventing progress towards advanced CRC	[139]
CRC patients	-NLRP3 expression in CRC↑ -The depth of tumor invasion, lymph node invasion, venous invasion and neural invasion in positive NLRP3 expression↑	-The survival of patients with positive NLRP3 expression -Targeting NLRP3 may be promising for targeted therapy of CRC, especially for mTORC1-targeted resistant patients	[140]
CRC patients	-TGF β, IL 1β, NF-κB, NLRP3 and caspase-1 protein and gene expression in CRC↑ -mRNA and protein levels of TGF-β, mature IL 1β, NF-κB and NLRP3 in patients with grade III ↑ -EMT markers N cadherin, vimentin and MMP 9 in CRC and also in grade III than grade I↑ -E-cadherin by the progression of CRC -NLRP3 protein level was inversely correlated with E-cadherin -NLRP3 protein level was positively correlated with IL 1β, active NF-κB, N cadherin, vimentin and MMP 9	-NLRP3 inflammasome activation contributed to the progression of CRC and is correlated with the EMT process	[141]
CRC cell lines and a subcutaneous tumor model	-NLRP3 in human CRC tissues↑ -Tumor size and invasion, lymph node metastasis, venous and neural invasion ↑ associated with NLRP-3 levels -knockdown of NLRP3 in CRC cells: CRC migration and growth in vitro ↓ and in vivo -knockdown of NLRP3 in CRC cells: Reversed EMT in vitro	-NLRP3 likely modulate CRC metastasis by EMT process activating -Potential therapeutic target	[142]
CRC patients	-Activated and functional caspase-1/IL-18 axis in tumor cells↑ -Drive a Th1/Tc1 response elicited by TILs expressing IL-18Rα	-Caspase-1/IL-18 axis targeting can improve the antitumor immunity in subgroups of CRC	[143]
Azoxymethane (AOM)/dextran sulfate sodium (DSS)-induced CAC mice	-Arctigenin administration: -NLRP3 inflammasome activation and fatty acid oxidation (FAO) metabolism in macrophages ↓ -The expression of carnitine palmitoyltransferase 1 (CPT1) ↓ -The acetylation of α-tubulin ↓ -NLRP3 complex formation ↓	-NLRP3 inflammasome assembly inhibition in macrophages due to downregulation of to FAO associated to the preventative effect of arctigenin against CAC	[144]

## Data Availability

Not applicable.
